# Effects of TNF-α, IL-1β and IL-2 on regulatory T cells in children with idiopathic nephrotic syndrome

**DOI:** 10.3389/fped.2026.1881956

**Published:** 2026-07-07

**Authors:** Fen-Fen Ni, Shi-Lei Jia, Cheng-Rong Li, Xiao-Jie Gao

**Affiliations:** Department of Nephrology, Shenzhen Children’s Hospital, Shenzhen, China

**Keywords:** idiopathic nephrotic syndrome, IL-1β, IL-2, TNF-α, treg cells

## Abstract

**Purpose:**

The pathogenesis of idiopathic nephrotic syndrome (INS) is unknown, but previous studies suggested that alterations of immune cells and cytokines have a role. We investigated the effects of multiple cytokines on the level of regulatory T cells (Tregs) in children with INS.

**Methods:**

Blood samples were collected from children with INS before and after glucocorticoid therapy and from age-matched healthy controls. The proportions of CD4^+^CD25^+^FOXP3^+^ Tregs were analyzed by flow cytometry. The plasma concentrations of TNF-α, IL-1β, and IL-2 were measured using a cytometric bead array. Real-time polymerase chain reaction (PCR) was used to detect the levels of Treg-associated factors, *TNF-*α, *IL-*1β, and *IL-*2-related signaling molecules in CD4^+^CD25^+^ T cells.

**Results:**

Compared with controls, children with active INS showed significantly decreased proportions of Tregs and reduced expression of *FOXP*3, *GITR*, and *CTLA-*4, accompanied by elevated plasma levels of TNF-α, IL-1β, and IL-2 (all *P* < 0.05). The expression of *TNFRII, HIF*1*α, PI*3*K, AKT*, and *mTOR* was significantly increased in the active INS group (all *P* < 0.05). Glucocorticoid treatment partially restored these abnormalities. Multiple linear regression indicated that TNF-α/TNFRII signaling was negatively correlated with *FOXP*3 expression and served as the main independent influencing factor.

**Conclusion:**

Patients with active INS have an increased level of TNF-α, which downregulated *FOXP*3, and led to overexpression of *TNFRII*. Aberrant signaling of the mTORC1/HIF*α* pathway in these patients may be mediated by an increased level of IL-1β. Aberrant signaling of the IL-2/PI3K pathway may be mediated by an increased level of IL-2, and this may contribute to downregulation of FOXP3^+^ Tregs.

## 1 Introduction

Idiopathic nephrotic syndrome (INS) is the most common glomerular disease in children and a main cause of chronic renal failure among children in China (1). INS is characterized by episodes of severe proteinuria and hypoalbuminaemia, damage to podocytes, foot process effacement, and alterations of the selective glomerular permeability barrier. Minimal change disease (MCD) is the most common form of INS, especially in patients younger than 10 years-old (2). Previous studies reported that these patients had obvious immune dysfunctions, including humoral immune disorders, abnormal secretion of cytokines, and T cell subset dysfunction [especially regulatory T cells (Tregs)] (3–9). Previous studies have documented not only Treg abnormalities but also broader T cell subset imbalances, including increased Th17 cells and altered Th1/Th2 ratios (3–7, 9), as well as B cell dysregulation in INS (8). These findings collectively suggest that INS involves a complex immune network rather than isolated Treg dysfunction. Although immune system changes may trigger and maintain INS (3–9), the causes of these immune dysfunctions remain unknown. Tregs are a distinct subset of T-lymphocytes that play a pivotal role in maintaining immune homeostasis and tolerance to self-antigens (10, 11). Several studies showed that the number of Tregs decreases and they have altered functional properties during the active phase of INS (8, 12–15), although the mechanisms causing Treg dysfunction remain unknown.

Certain cytokines can modify podocyte ultrastructure, although the cytokines responsible for development of proteinuria in INS are unknown (2–5). Alterations of multiple factors and cells may explain the pathology of INS, rather than the alteration of a single cytokine. TNF-α, IL-1β, and IL-2 are closely related to the function of Tregs, and may contribute to the formation of renal lesions in INS (8, 16–18). The cellular sources of elevated TNF-α, IL-1β, and IL-2 in active INS remain to be fully elucidated. However, previous studies suggest that activated peripheral T cells and infiltrating macrophages may contribute to cytokine production under inflammatory conditions (5, 7–9). Furthermore, podocytes themselves have been shown to express immune-related molecules such as CD80 and may respond to cytokine stimulation, suggesting a more active role in the local immune milieu (5, 17). The F0XP3 transcription factor is a reliable marker for Tregs (10, 11, 19). In addition, TNF-α-mediated stimulation of TNFRII at the Tregs surface leads to decreased *FOXP*3 expression (16). IL-1β signaling inhibits Treg differentiation via induction of *HIF-*1*α* transcription (17). IL-2 may affect Treg metabolism via PI3K signaling, leading to alterations in Treg lineage stability and suppressor function (18).

Thus, TNF-α, IL-1β, and IL-2 signaling pathways affect *FOXP*3 expression, but their association with Treg abnormalities in INS is unknown. We investigated the expression of TNF-α, IL-1β, IL-2, and associated signaling molecules in patients with INS to investigate the possible mechanisms responsible for FOXP3^+^ Tregs downregulation in these patients.

## 2 Materials and methods

### 2.1 Subjects

Forty children with INS (24 males, 16 females; median age: 38 months; age range: 12–122 months) from the inpatient pediatric population at Shenzhen Children's Hospital were enrolled from September 2015 to October 2016. All children met the following inclusion criteria: (*a*) Diagnosis of INS based on the 2009 Evidence-Based Guidelines for Diagnosis and Treatment of Common Pediatric Kidney Diseases in China; (*b*) steroid-sensitivity (i.e., negative urine protein within 4 weeks of treatment with a steroid and no other immunosuppressants); (*c*) no secondary kidney disease (secondary nephrotic syndrome, nephritic syndrome, congenital kidney disease, etc.); (*d*) no other systemic visceral syndrome; (*e*) provision of signed informed consent agreement from parents/guardians (20). These five criteria are concurrent requirements (all must be met), not mutually exclusive. The 40 children were divided into two groups: an active phase group (INS; 11 males, 9 females; median age: 33.5 months; age range: 12–80 months) and a remission group (INS-R; 13 males, 7 females; median age: 80.2 months; age range: 48–122 months). Children in the INS group were recently diagnosed with INS and were not yet under treatment; children in the INS-R group had INS and were under treatment, but did not use steroids for more than 4 weeks and had normal blood biochemical and urine test results. Twenty children of similar age (12 males, 8 females; median age: 31.3 months; age range: 18–112 months) who came to our hospital for physical examinations during the same period were enrolled as healthy controls (Ctrl). All participants had no history of infection within the previous 6 months, inflammatory conditions, or abnormal urinary sediment (casts or crystalluria). Supplementary Table S1 shows the demographic and clinical characteristics of all enrolled children. The INS-R group was older than the other two groups, but the groups had similar sex ratios. This study was approved by the Medical Ethics Committee of Shenzhen Children's Hospital (reference number 2015022). A paired longitudinal design was not used because some patients in the remission group lacked available pretreatment samples.

### 2.2 Blood samples

Venous blood was collected from enrolled children in EDTA-coated tubes. Whole blood was used directly for flow cytometric analysis. For plasma isolation, blood samples were centrifuged at 1,500× g for 10 min at 4 °C, and the supernatant plasma was aliquoted and stored at −80 °C until analysis by cytometric bead array (CBA). Peripheral blood mononuclear cells (PBMCs) were isolated from separate blood aliquots by Ficoll-Paque density gradient centrifugation (GE Healthcare, USA) for CD4⁺CD25⁺ T cell isolation (11363D, Dynal; Invitrogen, USA). Cell populations were considered pure (>97%) based on flow cytometry. Cell activity was assessed using the trypan blue exclusion assay, and dye uptake by 95% or more cells was considered an indicator of significantly decreased cell activity.

### 2.3 Total RNA extraction and cDNA synthesis

Total RNA was isolated from CD4^+^CD25^+^ T cells according to the manufacturer's instructions using the miRNeasy Mini Kit (Qiagen, Germany). The integrity of total RNA was confirmed by an average OD_260_/OD_280_ nm ratio of 1.98. cDNA was synthesized using oligo-dT primers and RevertAid^TM^ H Minus reverse transcriptase (Fermentas, Lithuania). Negative control samples (no first-strand synthesis) were prepared by performing reverse transcription in the absence of reverse transcriptase.

### 2.4 Lightcycler real-time PCR

Real-time PCR was performed on all 20 samples from each group (Ctrl, INS, INS-R). No samples were excluded due to low RNA quality or failed amplification. The cDNA levels of *FOXP*3, *GITR*, *CTLA*4, *TNFII*, *mTORC*1, *HIF*1*-α*, *PI*3*K*, *AKT*, and *mTOR* were quantitated by real-time PCR using the Quantitect™ SYBR green PCR Kit (Takara, Japan) and LightCycler® 2.0 (Roche Molecular Biochemicals, Switzerland). Supplementary Table S2 lists the primers used for the real-time PCR. The second derivative maximum method (Cp) was used for quantitation with LightCycler software version 3.5.30 (Roche Molecular Biochemicals). After normalization using Relative Quantification Software version 1.0 (Roche Molecular Biochemicals), the level of each target gene to that of *GAPDH* was reported.

### 2.5 Flow cytometry analysis of treg cells

Whole blood samples were processed by first incubating with anti-human CD4-FITC-A and anti-human CD25-APC (eBioscience, USA) at 4 °C for 30 min. Cells were then fixed, permeabilized, and stained with anti-human FOXP3-PE (eBioscience). Isotype controls were used for normalization and to confirm antibody specificity. Treg proportions were analyzed using a FACSCantoII cytometer equipped with FACS Diva version 6.1.3 software (Beckman Coulter, USA). The gating strategy was as follows: lymphocytes were first gated based on forward and side scatter, followed by gating on CD4⁺ lymphocytes, and then CD25⁺FOXP3⁺ cells were identified within the CD4⁺ gate. This gating strategy has been described previously (21).

### 2.6 CBA detection of plasma TNF-α, IL-1β and IL-2

The plasma levels of TNF-α, IL-1β, and IL-2 were measured using a CBA kit (eBioscience). All samples were measured in duplicate.

### 2.7 Statistical analysis

SPSS software for Windows version 22.0 was used for statistical analysis (SPSS Inc., USA). Data are expressed as means ± standard deviations. A one-way analysis of variance was used to compare multiple groups, pairwise comparison of groups was performed using the least-significant difference (LSD) test, and Student's *t*-test was used to compare 2 groups. Factor analysis and multiple linear regression analysis were used to identify correlations of different study parameters. *P*-values below 0.05 were considered significant.

## 3 Results

### 3.1 The proportion of CD4⁺CD25⁺FOXP3⁺ Tregs and related molecules is decreased in active INS

**Figure 1:**
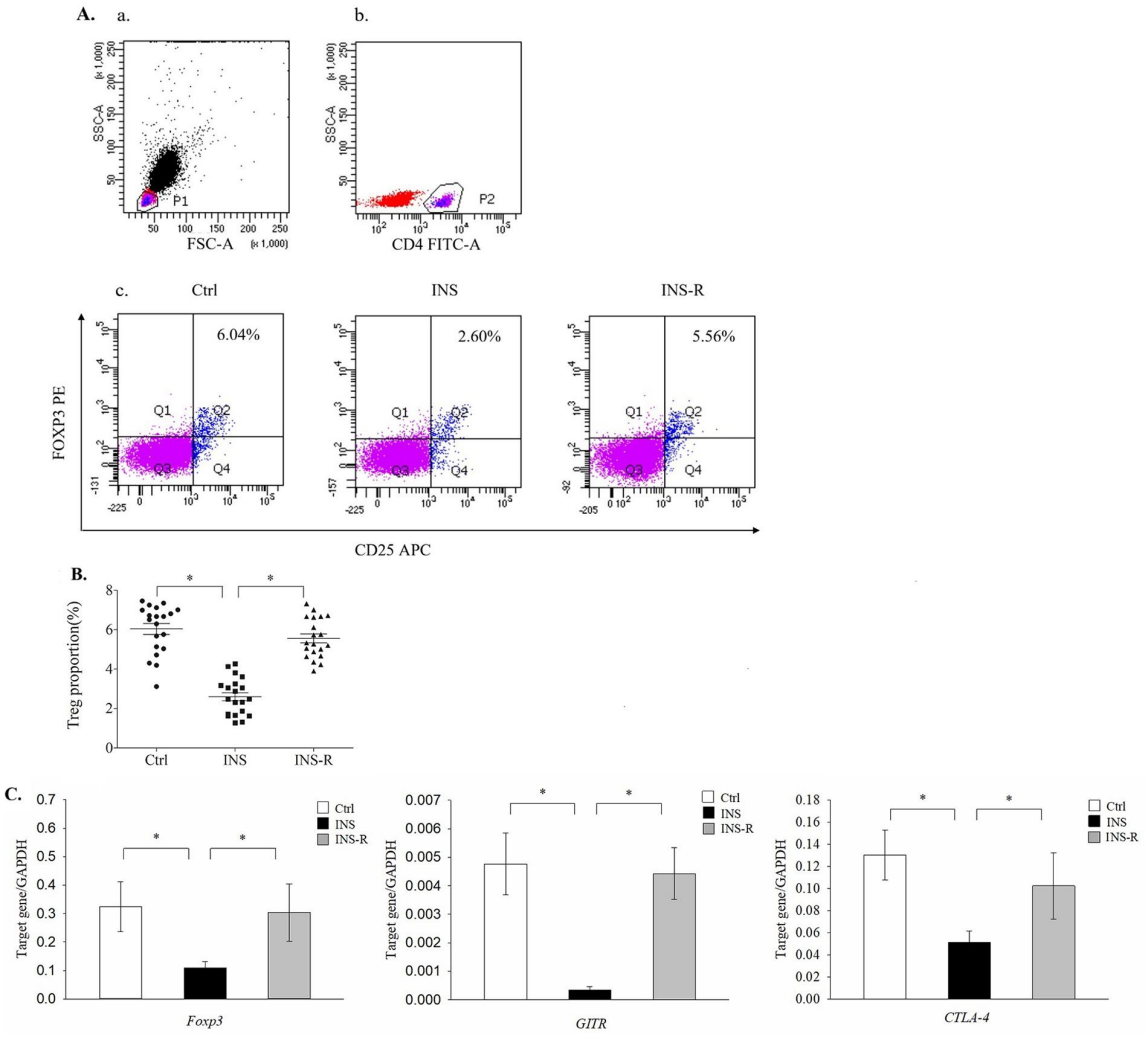
INS patients have altered expression of CD4^+^ CD25^+^Foxp3^+^ Tregs and Treg-related factors. **(A)** Flow cytometry of CD4^+^CD25^+^Foxp3^+^ Tregs. (a) Peripheral lymphocytes were gated in the dot plot of side scatter *vs.* P1. (b) CD4^+^ lymphocytes were gated in the dot plot of side scatter *vs.* P2. (c) Analysis of peripheral CD4^+^CD25^+^Foxp3^+^ Tregs in the different groups. **(B)** Proportions of CD4^+^ CD25^+^Foxp3^+^ Tregs in the different groups. **(C)** Expression of Treg-related factors in the different groups determined by real-time PCR using *GAPDH* as an endogenous reference. Data are shown as means ± SDs; statistical differences were determined using Student’s *t-*test; *P < 0.05. **P > 0.05; and Ctrl indicates healthy control, INS indicates idiopathic nephrotic syndrome, and INS-R indicates INS remission.

Analysis of Tregs from whole blood using flow cytometry (Figures 1A,B) indicated the INS group had a lower proportion of these cells than the Ctrl group (*P* < 0.05). The INS-R group had an intermediate proportion of Tregs, but this proportion was still significantly less than in the Ctrl group (*P* < 0.05). We also performed real-time PCR to evaluate the expression of *FOXP*3, *GITR*, and *CTLA-*4 in CD4^+^CD25^+^T cells (Figure 1C). The level of each mRNA was significantly lower in the INS group than in the Ctrl group and INS-R group (both *P* < 0.05).

### 3.2 TNF-*α*, IL-1*β*, and IL-2 are significantly elevated in active INS

**Figure 2:**
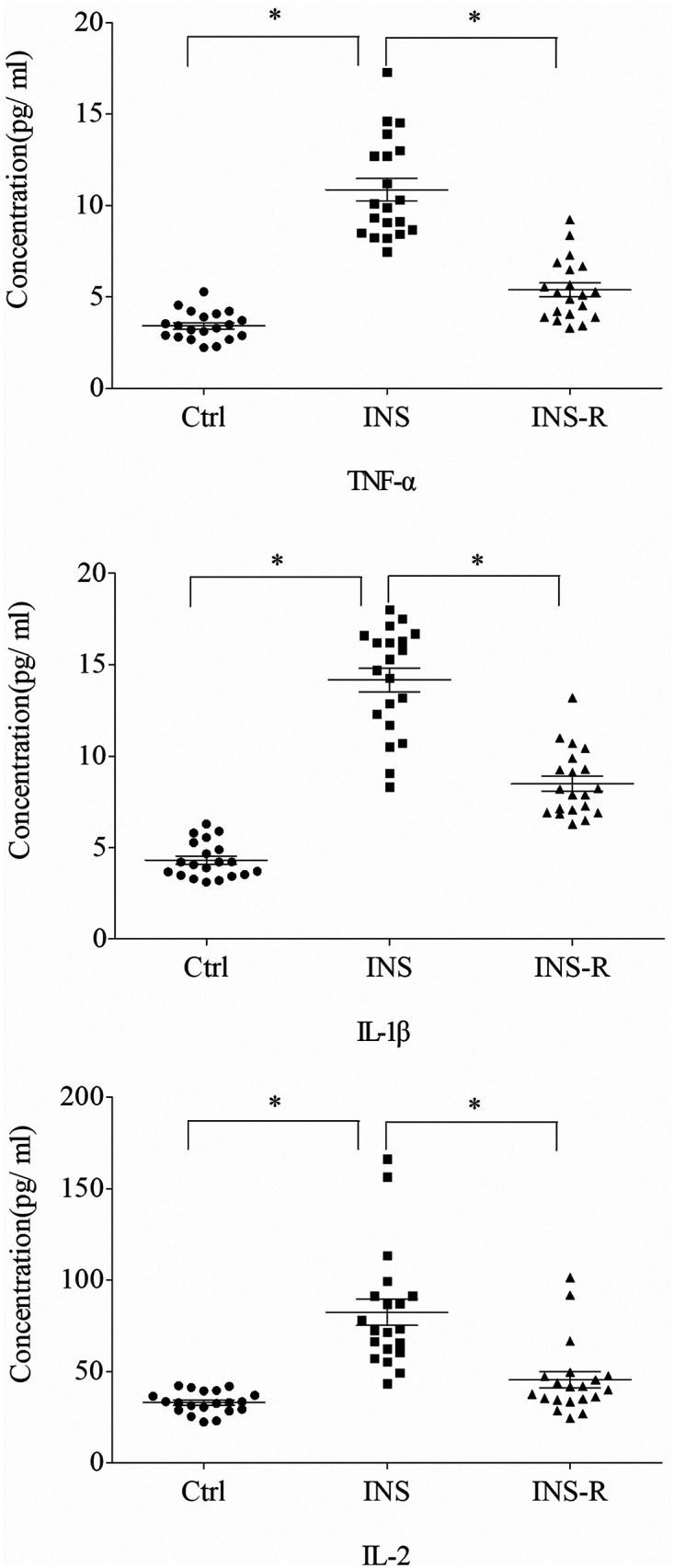
INS patients have increased plasma levels of TNF-α, IL-1β, and IL-2. Here and below: data are shown as means ± SDs; statistical differences were determined using Student's *t-*test; *P < 0.05. **P > 0.05; and Ctrl indicates healthy control, INS indicates idiopathic nephrotic syndrome, and INS-R indicates INS remission.

Previous studies showed that TNF-α, IL-1β, and IL-2 affected FOXP3 expression (8, 16–18). Because there is decreased expression of FOXP3 in the INS group, we determined the levels of these related cytokines using a cytometric bead array (Figure 2). The results indicated increased plasma levels of each cytokine in the INS group relative to the Ctrl group (all *P* < 0.05) and relative to the INS-R group (all *P* < 0.05). The INS-R group had slightly (but not significantly) higher levels of each cytokine than the Ctrl group.

### 3.3 The expression of TNFRII is significantly increased in children with active INS

**Figure 3:**
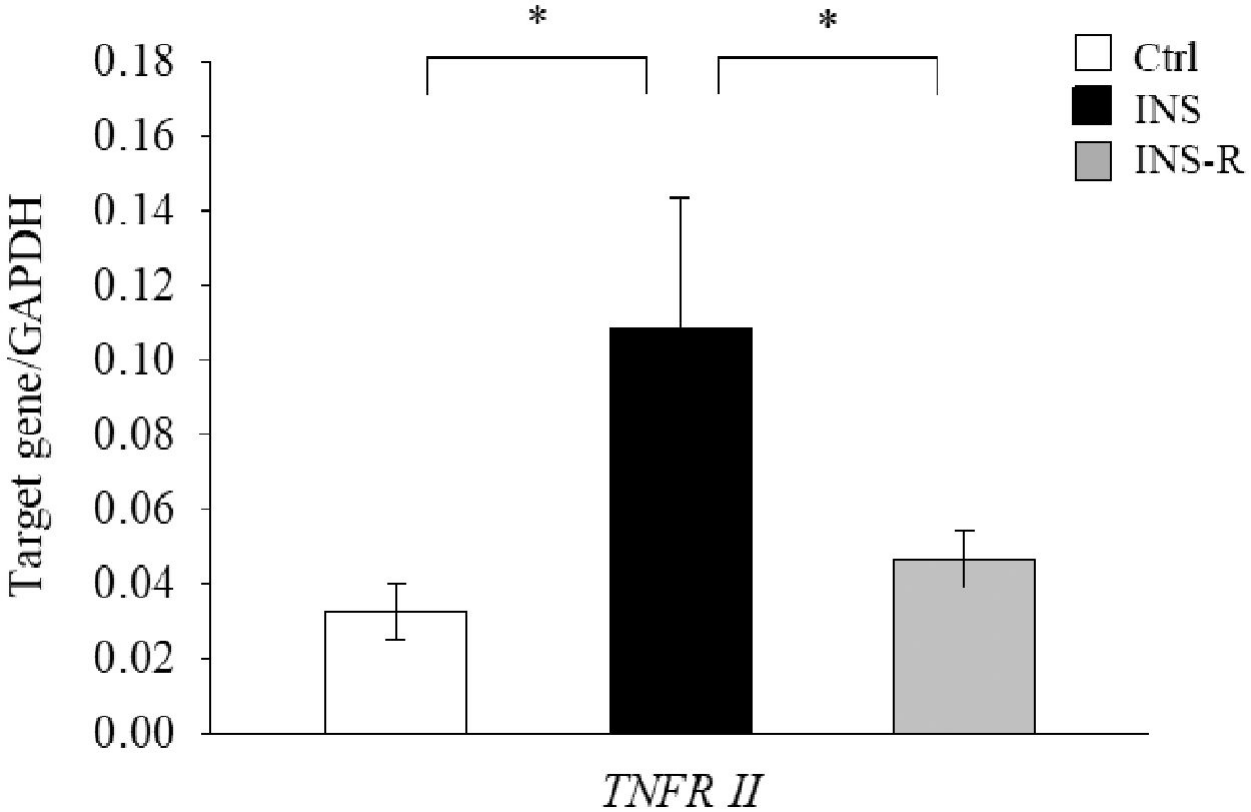
INS patients have increased levels of a TNF-α-related signaling molecule (*TNFRII*). Here and below: expression was determined by real-time PCR and is presented relative to *GAPDH* (endogenous reference). Data are shown as means ± SDs; statistical differences were determined using Student's *t-*test; *P < 0.05. **P > 0.05; and Ctrl indicates healthy control, INS indicates idiopathic nephrotic syndrome, and INS-R indicates INS remission.

TNF-α stimulation of TNFRII at the Tregs surface decreases FOXP3 expression (22–24). Thus, we used real-time PCR to measure the expression of TNFⅡ in the different groups (Figure 3). The results indicated higher expression of TNFⅡ in the INS group than in the Ctrl group and the INS-R group (both *P* < 0.05). As above, the INS-R group had slightly (but not significantly) higher level of TNFII than the Ctrl group.

### 3.4 The expression of mTORC1 and HIF1*α* is upregulated in active INS

**Figure 4:**
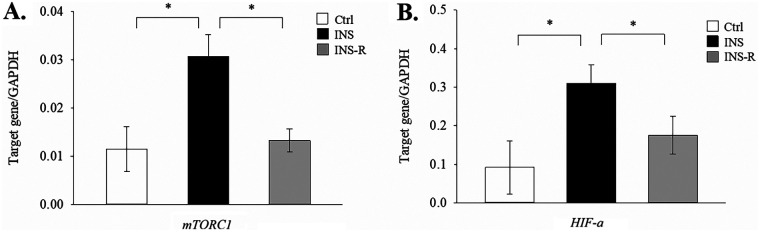
INS patients have increased levels of IL-1β-related signaling molecules (*mTORC*1 and *HIF-*1*a*). **(A)** The expression of mTORC1; **(B)** The expression of HIF1α. Here and below: expression was determined by real-time PCR and is presented relative to *GAPDH* (endogenous reference). Data are shown as means ± SDs; statistical differences were determined using Student's *t-*test; *P < 0.05. **P > 0.05; and Ctrl indicates healthy control, INS indicates idiopathic nephrotic syndrome, and INS-R indicates INS remission.

IL-1β inhibits Treg differentiation via induction of mTORC1/HIF-1*α* signaling (17). Thus, we used real-time PCR to determine the levels of *mTORC*1 and *HIF-*1*α* in the different groups (Figures 4A,B). The results indicated higher expression of each mRNA in the INS group than in the Ctrl group and the INS-R group (both *P* < 0.05). As above, the INS-R group had slightly (but not significantly) higher levels of each mRNA than the Ctrl group.

### 3.5 The expression of PI3K, AKT, and mTOR is significantly upregulated in active INS

**Figure 5:**
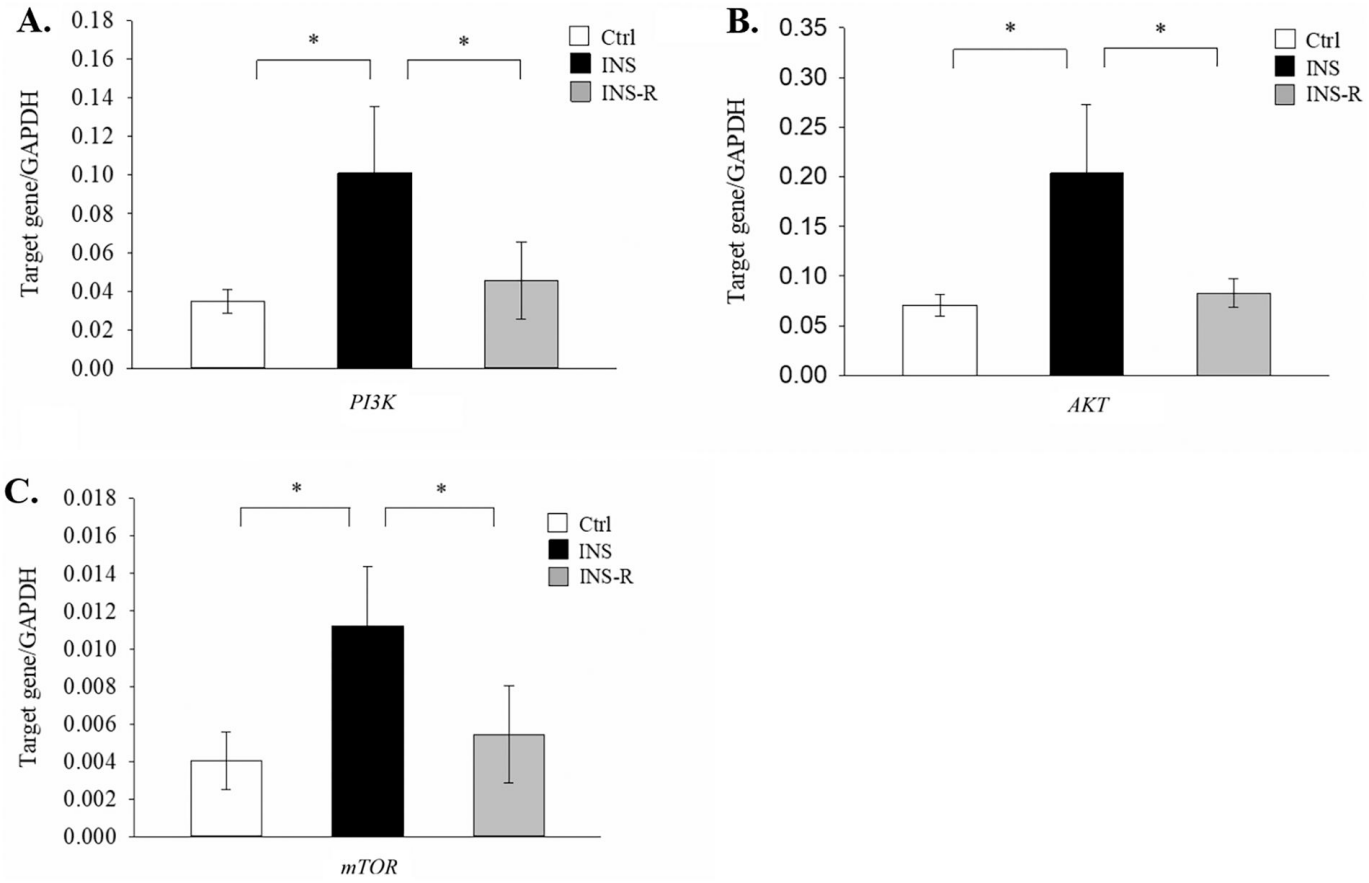
INS patients have increased expression of IL-2-related signaling molecules. **(A)** The expression of PI3K; **(B)** The expression of Akt; **(C)** The expression of mTOR. Here and below: expression was determined by real-time PCR and is presented relative to *GAPDH* (endogenous reference). Data are shown as means ± SDs; statistical differences were determined using Student's *t-*test; *P < 0.05. **P > 0.05; and Ctrl indicates healthy control, INS indicates idiopathic nephrotic syndrome, and INS-R indicates INS remission.

IL-2 can affect Treg metabolism via the PI3K/AKT/mTOR signaling pathway (18). We therefore used real-time PCR to determine the levels of three IL-2-related signaling molecules (Figures 5A–C). The results indicated significantly higher levels of *PI*3*K*, *AKT*, and *mTOR* in the INS group relative to the Ctrl group and the INS-R group (all *P* < 0.05). As above, the INS-R group had slightly (but not significantly) higher levels of each mRNA than the Ctrl group.

### 3.6 TNF-*α*/TNFRII signaling is negatively correlated with FOXP3 expression and acts as the main independent regulatory factor

The results above indicated a relationship of cytokine signaling pathways with the expression of *FOXP*3 in patients with INS. Although each of the measured components in these pathways may be colinear, it is also possible that the outputs from the different pathways may vary independently. We cannot directly measure whether certain common factors are independent of each other, because each common factor has its own significance that does not overlap with other factors. Therefore to better understand the relationship of *FOXP*3 level with TNF-α, IL-1β, and IL-2-related signaling molecules in INS, we used data from the INS group for factor analysis and multiple linear regression analysis.

The results of the factor analysis (Supplementary Table S3) indicated that the factor-1 explained 15.93% of the variance and had an eigenvalue of 1.48; factor-2 explained 24.716% of the variance and had a eigenvalue of 2.482; and factor-3 explained 37.208% of the variance and had an eigenvalue of 3.044. Factor-1 mainly controls *TNF-*α and *TNFII* expression, factor-2 mainly controls *IL-*1β and *HIF*1*-α* expression, and factor-3 mainly controls *IL-*2, *PI*3*K*, *AKT* and *mTOR* expression. Based on the factor score coefficients calculated for each sample's predictive value affecting *FOXP*3 expression, we generated the following predictive model:

Factor-1=0.822×(TNF-α+0.812)×TNFII
Factor-2=0.951×(IL-1β+0.914)×(mTORC1-α+0.825)×HIF1-α
Factor-3=0.625×(IL-2+0.955)×(PI3K+0.967)×(AKT+0.868)×mTOR

The results of multiple linear regression analysis (Supplementary Table S4) indicated that the TNF-*α*/TNFRII signaling pathway had a significant negative association with *FOXP*3 mRNA levels (standardized Beta = –0.797, R² = 0.659, *P* < 0.05), suggesting that increased TNF-*α*/TNFRII signaling is associated with reduced *FOXP*3 expression. In contrast, Factor-2 (IL-1*β*/HIF-1*α*) and Factor-3 (IL-2/PI3K/AKT/mTOR) did not reach statistical significance in the multivariate model (*P* > 0.05), indicating that TNF-*α*/TNFRII may be the predominant pathway affecting *FOXP*3 in this cohort.

## 4 Discussion

A large body of evidence has demonstrated that immune system dysregulation may play a crucial role in INS (3–9), although there are many uncertainties in the pathogenesis of this disease. Some researchers hypothesized that INS may be caused by abnormal secretion of cytokines and T cell subset dysfunction, especially dysfunction of Tregs (3–9). Tregs consist of two subsets: natural Tregs (nTregs), which are derived from the thymus, and induced Tregs (iTregs), which are generated in the peripheral system from CD4^+^CD25^−^ T cells or induced from naive CD4^+^ T cells *in vitro* (10–12). Recently, Shimada et al. proposed a “two-hit” hypothesis for minimal change disease, in which there is induction of CD80 (or B7-1) and Treg dysfunction, with or without impaired autoregulatory function of the podocytes (23). Treg dysfunction could transform massive transient proteinuria into persistent proteinurea, leading to podocyte injury, and eventually minimal change nephrotic syndrome (5, 8, 12). The importance of the association between Tregs and nephrotic syndrome is highlighted by the presence of immune dysregulation. However, the reason why children with INS have Treg impairment and/or podocyte dysfunction is unknown. FOXP3 is a transcription factor expressed in Tregs, and a key regulator of their differentiation and suppressor function (10, 11, 19). Several studies have reported that the number and function of Tregs are reduced in active INS, and that changes are related to disease development and the response to glucocorticoids (8, 12, 15). Consistent with previous findings, our study demonstrated that Tregs and the expression of Treg-related factors (*FOXP*3, *GITR*, *CTLA-*4) were significantly lower patients with active INS. After glucocorticoid treatment, there was some restoration of Treg numbers expression of these related factors, but the mechanism remains unclear.

INS is believed to be a renal manifestation of T-cell dysregulation, in which cytokines act as modifiers of podocyte ultrastructure (2, 5, 8). However, numerous studies have failed to identify the cytokine responsible for the development of proteinuria in INS. It is likely that a single cytokine is not responsible for the pathogenesis, and that a complex set of factors and cells better explains the pathogenesis. TNF-α and IL-1β are powerful pro-inflammatory cytokines, and are involved in the processes of immune inflammation and damage of glomerular cells (7–9). There is evidence that abnormal expression of TNF-α and IL-1β function in the pathogenesis of lupus nephritis, diabetic nephropathy, and other autoimmune diseases (24–26). TNF-α, IL-1β, and IL-2 are closely related to Tregs (10, 16–18), and are considered possible pathogenic factors underlying the mechanisms of renal lesions in INS (8, 9, 12, 23). Potential mechanisms for increased cytokine levels include systemic immune activation, T cell dysregulation, local renal inflammation, and podocyte injury, which may form a proinflammatory feedback loop that aggravates Treg dysfunction(5.8,12). However, it is not clear whether aberrant expression of these cytokines in INS patients results in decreased pools of FOXP3^+^ Tregs.

Our results indicated that the plasma levels of TNF-α, IL-1β, and IL-2 were significantly increased during the active phase of INS. We performed factor analysis to better understand the relationship between aberrant expression of pro-inflammatory cytokines and changes in the expression of *FOXP*3 and other factors in INS patients. The results indicated that factors controlling TNF-α, IL-1β, and IL-2 expression also influenced *FOXP*3 expression. Therefore, we propose that the decreased population of FOXP3^+^ Tregs in INS patients may be related to the over-expression of plasma TNF-α, IL-1β, and IL-2.

We investigated the mechanisms responsible for abnormal cytokine expression in children with active INS. Previous studies showed that TNF-α inhibits the suppressive function of FOXP3^+^ Tregs, particularly by suppressing inflammation (8, 12, 16, 27). Other research showed that TNF-α signaling via TNFRII downregulates *FOXP*3 expression in humans in naturally-occurring and adaptive Tregs, and this leads to inhibition of their suppressive activity (16, 22, 28). The effect of TNF-α/TNFRII signaling on Treg abnormalities in active INS requires further study. We found that *TNFRII* levels were significantly greater in children with active INS than in healthy controls, but was slightly lower for children in remission. Multiple linear regression analysis showed that TNF-α/TNFRII expression was mainly governed by factor-1, which had a statistically significant negative correlation with FOXP3 expression (Beta = −0.797). Therefore, we speculate that aberrant TNF-α/TNFRII signaling might contribute to the down-regulation of FOXP3^+^ Tregs in active INS.

IL-1β activates mTORC1 and upregulates HIF-1*α* (17, 18). HIF-1*α* mainly has a detrimental effect on the function and stability of Tregs, and is necessary for the development and suppressive function of Tregs (10, 17, 19). Our data showed that children with active INS had elevated levels of *mTORC*1 and *HIF-*1*α*. After glucocorticoid therapy, the levels of *mTORC*1 and *HIF-*1*α* declined slightly. We speculate that aberrant mTORC1/HIF-1*α* signaling resulting from an increased level of IL-1β might explain the down-regulation of FOXP3^+^ Tregs in active INS. Our data support this hypothesis, but multivariate regression analysis indicated that factor-2, which mainly controls the IL-1β/mTORC1/HIF-1*α* signaling pathway, had no significant effect on *FOXP*3 expression. We therefore hypothesize that other heretofore unidentified factors affect IL-1β/mTORC1/HIF-1*α* signaling.

The PI3K/Akt/mTOR cascade constitutes a major signaling pathway downstream of IL-2, and is closely tied to cellular metabolism (10, 17, 18). Glycolysis is primarily activated in Tregs through mTOR, and tends to suppress *FOXP*3 expression and Treg lineage stability (10, 17, 18). We found that the expression of *PI*3*K, AKT* and *mTOR* were significantly increased in children with active INS relative to healthy controls, but their levels were slightly less in children who were in remission. Thus, it is feasible that aberrant signaling of the PI3K/Akt/mTOR pathway may be mediated by an increased level of IL-2, and thus contribute to the downregulation of FOXP3^+^ Tregs in active INS. However, our multivariate regression analysis indicated that factor-3, which mainly controls the IL-2/PI3K signaling pathway, had no effect on *FOXP*3 expression. As above, we speculate that other unidentified factors affect IL-2/PI3K signaling.

Glucocorticoids represent the mainstay of treatment for INS and might affect the pathogenesis of this disease (2, 8, 20, 29). Previous research reported that intravenous injection of methylprednisolone in children with lupus nephritis and severe proteinuria led to an increased level of Treg cells in the peripheral blood and relief of proteinuria (30–32). These results indicate that the increase of Tregs plays an important role in relieving proteinuria. Glucocorticoids can protect against some immune-mediated diseases by increasing the number of peripheral Treg cells, but the mechanism is unknown. Our results indicated that patients with active-phase (first-onset) INS had higher levels of plasma TNF-α, IL-1β, IL-2 than those in remission, and that remission was also associated with partial normalization of pathways regulated by these cytokines and of circulating Tregs. Although it is unclear how glucocorticoid treatment reduced the plasma levels of TNF-α, IL-1β, IL-2, restored cytokine-related signaling, and normalized the circulating levels of FOXP3^+^ Tregs, our results provide new insights into the immunoregulatory mechanism of glucocorticoids in the treatment of INS.

The TNF-*α*/TNFRII-mediated downregulation of FOXP3 has also been reported in rheumatoid arthritis and systemic lupus erythematosus (16, 28), suggesting that this mechanism may be a common pathway of Treg dysfunction in autoimmune diseases rather than a feature specific to INS. Similarly, IL-1*β*/mTORC1/HIF-1*α* and IL-2/PI3K/AKT/mTOR signaling have been implicated in Treg instability in various inflammatory conditions. Therefore, the phenomena observed in this study may represent a general immune regulatory pattern shared among immune-mediated glomerular diseases, although the relative contribution of each pathway may differ by disease context.

Our findings raise the possibility that plasma TNF-α, IL-1β, and IL-2 may serve as potential auxiliary biomarkers for evaluating disease activity and monitoring therapeutic responses in pediatric INS. All three cytokines were significantly elevated in active disease and partially normalized after glucocorticoid-induced remission. Their levels also correlated with the degree of Treg reduction. Prospective studies are needed to evaluate whether baseline cytokine levels predict steroid resistance or relapse. If validated, these cytokines might help monitor treatment response and guide immunosuppressive therapy.

In summary, the down-regulation of FOXP3^+^ Tregs may be associated with aberrant signaling of the TNF-α/TNFRII, IL-1β/mTORC1/HIF-1*α*, and IL-2/PI3K/Akt/mTOR pathways in patients with active INS. After anti-inflammatory or immunomodulatory treatment with glucocorticoids, there is some restoration and normalization of cytokine signaling and Treg cells. In addition, abnormal TNF-α signaling is the main factor affecting FOXP3^+^ Tregs. Thus, the level of TNF-α may indicate the disease state and could potentially be used to monitor the efficacy of INS treatments. Furthermore, This study has some limitations. First, *in vitro* functional experiments were not performed due to limited cell numbers from pediatric samples; thus, direct regulatory effects of cytokines on Tregs could not be confirmed. Second, our data do not distinguish between reduced Treg survival, impaired differentiation, or functional exhaustion, all of which could contribute to the decreased Treg pool observed in active INS. Third, a notable age disparity existed across the three groups, and participants in the INS-R group were older than those in the Ctrl and active INS groups. Given that age affects baseline Treg frequencies and cytokine profiles in children, we conducted an analysis of covariance (ANCOVA) using age as a covariate to mitigate this confounding factor. After age adjustment, all key intergroup differences remained statistically significant (data not shown). We fully recognize this limitation. Future longitudinal studies with paired pre- and post-treatment specimens from the same individuals are needed to reduce inter-individual variability, including age-associated impacts. Fourth, we analyzed total FOXP3^+^ Tregs without distinguishing nTreg and iTreg subsets. TNF-α, IL-1β, and IL-2 may differentially regulate these subsets, which requires further investigation. Finally, we did not examine other Treg-like populations, including Tr1, Th3, CD8⁺CD28⁻, and Qa-1 restricted T cells. The role of these populations in INS needs further study.

## Data Availability

The original contributions presented in the study are included in the article/Supplementary Material, further inquiries can be directed to the corresponding author.
