# Supplementary material for: Effects of TNF-α, IL-1β and IL-2 on regulatory T cells in children with idiopathic nephrotic syndrome
Source: Front Pediatr. 2026 Jul 7;14:1881956. doi: 10.3389/fped.2026.1881956 (PMC13386491; doi:10.3389/fped.2026.1881956)
Supplement: Supplementary file 1 [file Table1.doc]

**Supplementary Table 1. Characteristics of the control, INS, and INS-R groups.**

| Group Ctrl | | Active INS | Remission INS |
| --- | --- | --- | --- |
| Number of subjects | 20 | 20 | 20 |
| Male/female, n (%) | 12/08 (60) | 11/09 (55) | 13/07 (65) |
| Age in months, median (range) | 31.3 (18–112) | 33.5 (12–80) | 80.2 (48–122) |
| Urinary proteins, g/24h | 0.041 ± 0.01 | 2.27 ± 0.28^a b^ | 0.052 ± 0.01 |
| Serum albumin, g/L | 46.79 ± 1.01 | 17.35 ± 0.67^a b^ | 43.23 ± 1.23 |
| Total cholesterol, mg/dL | 3.67 ± 0.16 | 9.97 ± 2.14^a b^ | 3.88 ± 0.22 |
| Triglycerides, mg/dL | 0.78 ± 0.01 | 2.37 ± 0.02^a b^ | 0.89 ± 0.01 |
| Serum uric acid, μmol/L | 290.2 ± 38.9 | 336 ± 22.5 | 269.5 ± 29.6 |
| Urea nitrogen, mmol/L | 3.92 ± 0.40 | 4.48 ± 0.61 | 4.77 ± 0.52 |
| Serum creatinine, μmol/L | 30.33 ± 4.23 | 33.72 ± 5.12 | 35.43 ± 6.01 |
|  |  |  |  |

Data are shown as means ± SEMs. Normal values: urinary protein: <0.15 g/24h, serum albumin: 35–55 g/L, total cholesterol <4.4 mg/dL, triglycerides <1.7 mg/dL, serum uric acid: 90–420 μmmol/L, urea nitrogen: 1.5–7.0 mmol/L, serum creatinine: 21-65 μmmol/L.

^a^ P<0.01, compared with the Ctrl group; ^b^ P<0.01, compared with the INS-R group
