# Supplementary material for: Effects of TNF-α, IL-1β and IL-2 on regulatory T cells in children with idiopathic nephrotic syndrome
Source: Front Pediatr. 2026 Jul 7;14:1881956. doi: 10.3389/fped.2026.1881956 (PMC13386491; doi:10.3389/fped.2026.1881956)
Supplement: Supplementary file 2 [file Table2.doc]

**Supplementary Table 2. Primers used for real-time PCR.**

| Gene | Primer sequence | Annealing temp. (°C) | Product size, bp |
| --- | --- | --- | --- |
| *FOXP3* | Sense:  5’-GGAAAGGAGGATGGACGAAC-3’  Antisense:  5’-GCAGGCAAGACAGTGGAAAC-3’ | 56 | 122 |
| *GITR* | Sense:  5’-ACACGCACTTCACCTGGGTCG-3’  Antisense:  5’-TGTGCCATGCTCGGGTTTCA-3’ | 56 | 129 |
| *CTLA-4* | Sense:  5’-GTCCGGGTGACAGTGCTTCG-3’  Antisense:  5’-CCAGGTAGTATGGCGGTGGG-3’ | 56 | 220 |
| *TNFRII* | Sense:  5’-CCGATTACCGGCGCATCACG -3’  Antisense:  5’-TGGGTCCCGAGGCCATCTTCAC-3’ | 56 | 186 |
| *mTORC1* | Sense: 5’-CAGAAACCCTAAAGCTGCATTGTAA-3’  Antisense: 5’-GTCTGTTCAGTGACCTACAAACACC-3’ | 60 | 180 |
| HIF-1α | Sense:  5’-GAAGTGTACCCTAACTAGCCG-3’  Antisense: 3’-TTCACAAATCAGCACCAAGC-5’ | 56 | 163 |
| *PI3K* | Sense:  5’-AGCATTGGGACCTCACATTACACA-3’  Antisense: 5’-ACTGGAAACACAGTCCATGCACATA-3’ | 60 | 138 |
| *AKT* | Sense:  5’-CTTGCTTTCAGGGCTGCTCA-3’  Antisense:  3’-TACACGTGCTGCCACACGATAC-5’ | 60 | 117 |
| *mTOR* | Sense:  5’-ATGCTTGGAACCGGACCTG-3’  Antisense:  5’-TCTTGACTCATCTCGGAGTT-3’ | 60 | 173 |
| *GAPDH* | Sense:  5’-CAAGAAGGTGGTGAAGCAGG-3’  Antisense:  5’-AGGTGGAGGAGTGGGTGTCG-3’ | 54–60 | 110 |
