# Supplementary material for: Effects of TNF-α, IL-1β and IL-2 on regulatory T cells in children with idiopathic nephrotic syndrome
Source: Front Pediatr. 2026 Jul 7;14:1881956. doi: 10.3389/fped.2026.1881956 (PMC13386491; doi:10.3389/fped.2026.1881956)
Supplement: Supplementary file 3 [file Table3.doc]

**Supplementary Table 3. Factor analysis of components affecting *FOXP3* expression in INS.**

| Factor | % variance | cumulative % | Component (extraction factor) | | | |
| --- | --- | --- | --- | --- | --- | --- |
|  |  |  | Factor-1  loadings | Factor-2  loadings | Factor-3  loadings | Communalities |
| *TNF-α* | 15.930 | 15.930 | 0.822 |  |  | 0.710 |
| *TNFRII* |  |  | 0.812 |  |  | 0.663 |
| *IL-1β* | 24.716 | 40.646 |  | 0.951 |  | 0.930 |
| *mTORC1* |  |  |  | 0.914 |  | 0.840 |
| *HIF-1α* |  |  |  | 0.825 |  | 0.771 |
| *IL-2* | 37.208 | 77.854 |  |  | 0.625 | 0.400 |
| *PI3K* |  |  |  |  | 0.955 | 0.922 |
| *AKT* |  |  |  |  | 0.967 | 0.941 |
| *mTOR* |  |  |  |  | 0.868 | 0.830 |
| Eigenvalue |  |  | 1.480 | 2.482 | 3.044 |  |
