# Supplementary material for: Effects of TNF-α, IL-1β and IL-2 on regulatory T cells in children with idiopathic nephrotic syndrome
Source: Front Pediatr. 2026 Jul 7;14:1881956. doi: 10.3389/fped.2026.1881956 (PMC13386491; doi:10.3389/fped.2026.1881956)
Supplement: Supplementary file 4 [file Table4.doc]

**Supplementary Table 4. Multiple linear regression analysis of the relationship of *FOXP3* expression with Factor 1, Factor 2, and Factor 3**

| Group | Beta  (standardized  regression  coefficient) | t | *P* |
| --- | --- | --- | --- |
| Factor-1 | -0.797 | -5.189 | <0.001 |
| Factor-2 | -0.058 | 0.385 | 0.705 |
| Factor-3 | -0.073 | 0.474 | 0.642 |

The factor score coefficients for each sample were used to predict each factor’s effect on *FOXP3* expression. Then, a residual analysis test was used to test whether each factor met the conditions for multivariate linear regression analysis. Finally, stepwise regression analysis was used to assess the relationship of *FOXP3* expression with each factor.
